# Supplementary material for: PEDOT:PSS-Coated Polybenzimidazole Electroconductive Nanofibers for Biomedical Applications
Source: Polymers (Basel). 2021 Aug 19;13(16):2786. doi: 10.3390/polym13162786 (PMC8401200; doi:10.3390/polym13162786)
Supplement: Supplementary file 1 [file polymers-13-02786-s001.zip › polymers-1324756-supplementary.pdf]

Supplementary information

PEDOT:PSS-Coated Polybenzimidazole Electroconductive Nanofibers for Biomedical Applications

Laura Sordini [1,2,3], João C. Silva [1,2,4], Fábio F. F. Garrudo [1,2,3,5], Carlos A. V. Rodrigues [1,2], Ana C. Marques [6], Robert J. Linhardt [5], Joaquim M. S. Cabral [1,2], Jorge Morgado [3,*] and Frederico Castelo Ferreira [1,2,*]

[1] iBB—Institute for Bioengineering and Biosciences and Department of Bioengineering, Instituto Superior Técnico, Universidade de Lisboa, Av. Rovisco Pais, 1049-001 Lisboa, Portugal; laura.sordini@tecnico.ulisboa.pt (L.S.); joao.f.da.silva@tecnico.ulisboa.pt (J.C.S.); fabio.garrudo@tecnico.ulisboa.pt (F.F.F.G.); carlos.rodrigues@tecnico.ulisboa.pt (C.A.V.R.); joaquim.cabral@tecnico.ulisboa.pt (J.M.S.C.)

[2] Associate Laboratory i4HB—Institute for Health and Bioeconomy, Instituto Superior Técnico, Universidade de Lisboa, Av. Rovisco Pais, 1049-001 Lisboa, Portugal

[3] Instituto de Telecomunicações and Department of Bioengineering, Instituto Superior Técnico, Universidade de Lisboa, Av. Rovisco Pais, 1049-001 Lisboa, Portugal

[4] CDRSP – Centre for Rapid and Sustainable Product Development, Polytechnic Institute of Leiria, Rua de Portugal-Zona Industrial, 2430-028 Marinha Grande, Portugal

[5] Center for Biotechnology & Interdisciplinary Studies, Department of Chemistry & Chemical Biology, Rensselaer Polytechnic Institute, Troy, NY 12180, USA; linhar@rpi.edu

[6] CERENA, Departament of Chemical Engineering, Instituto Superior Técnico, Universidade de Lisboa, Avenida Rovisco Pais, 1049-001 Lisboa, Portugal; ana.marques@tecnico.ulisboa.pt

* Correspondence: jmfmorgado@tecnico.ulisboa.pt (J.M.); frederico.ferreira@tecnico.ulisboa.pt (F.C.F.)

1. ATR-FTIR and TGA analysis

1.1. Materials and Methods and Data processing:

ATR-FTIR spectra of PBI containing samples presented in **Figure S1A** were normalized to the common at peak 1443 cm^{-1} and experimental conditions to obtaining those spectra can be found on the main manuscript.

Thermogravimetric analysis (TGA) of the fiber mats was studied under controlled air atmosphere (20% oxygen), with a flow of 100 $mL.min^{-1}$, using a computer-controlled Hitachi STA 7200 (Chiyoda, Tokyo, Japan) at a constant heating rate of 5 $°C.min^{-1}$ up to 600 °C. The onset temperature of degradation and corresponding mass losses were calculated for each sample using the thermograms and corresponding first derivative curves.

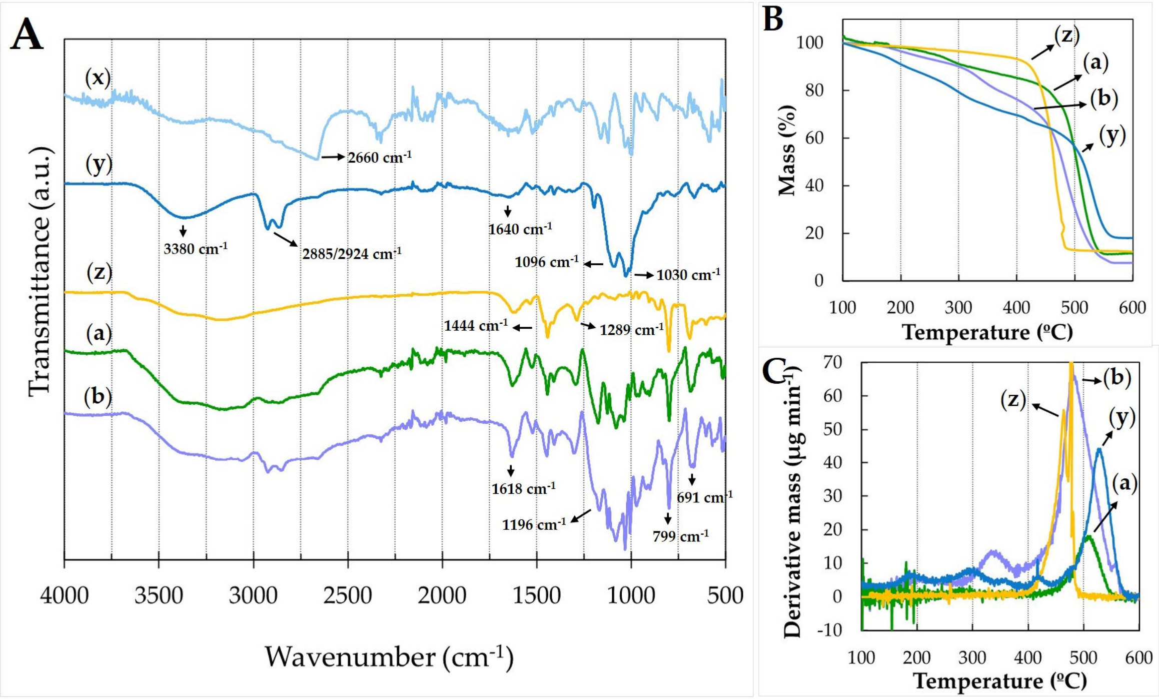

Figure S1. (**A**) ATR-FTIR spectra, (**B**) Thermogravimetric analysis (TGA) (20 % oxygen) and (**C**) respective first derivative of the various samples produced in this work: (**x**) PEDOT:PSS pellet, (**y**) PEDOT:PSS:GOPS pellet, (**z**) pristine electrospun PBI fibers and cross-linked PEDOT:PSS-coated PBI fibers obtained either by (a) spin coating or (b) dip coating.

1.2. Brief Results and Discussion

ATR-FTIR analysis of pellets: The ATR-FTIR spectra of PEDOT:PSS and PEDOT:PSS pellets with GOPS are depicted in **Figure S1A** (spectra **x** and **y**, respectively). PEDOT:PSS spectrum (**Figure S1Ax**) presents several characteristic peaks in the region 500-1800 cm^{-1} [1,2], namely at 665 cm^{-1} and 860 cm^{-1} (thiophene ring, ν (C-S) and δ (C-H)), and at 1000 cm^{-1}, 1026 cm^{-1}, 1123 cm^{-1} and 1158 cm^{-1} (cyclic ethers, C-O stretching in PEDOT and sulfoxide in PSS) and at 1300 cm^{-1} (C-C) and 1519 cm^{-1} (C=C), from the thiophene ring. The peak at 2660 cm^{-1} is possibly due to alkane C-H bonds stretching [1]. The ATR-FTIR spectrum of PEDOT:PSS pellets with GOPS (**Figure S1Ay**), besides the presence of the PEDOT:PSS peaks, evidences the presence of GOPS reaction products. GOPS may react via

condensation between Si-OCH_3 groups or via ring opening polymerization of the epoxy (oxirane) group. Peaks at 1096 cm^{-1} and 1030 cm^{-1} are attributed to the Si-O-Si stretching vibrations indicating the formation of a siloxane network via condensation of the methoxy groups [3]. The presence of unreacted methoxy group Si-O-CH_3 is identified by the peak at 1196 cm^{-1} and possibly also at 1096 cm^{-1}. The absence of characteristic bands from the GOPS´s epoxy (oxirane) group, namely at 1250 cm^{-1} [4], suggests that the majority of the oxirane groups have reacted by ring opening polymerization, during the cross-linking step, possibly by reaction with the sulfonate group of PSS. A double peak is found at 2855/2924 cm^{-1}, attributed to GOPS C-H bond stretching. The broad band centered at ca. 3380 cm^{-1} is related to the presence of OH groups, from residual water and/or from Si-OH groups.

ATR-FTIR of coated fibers: The spectra of the cross-linked PEDOT:PSS-coated PBI fiber samples obtained by spin and dip coating are depicted in **Figure S1Aa** and **S1Ab**, respectively. Again, ATR-FTIR spectra of PBI containing samples presented in **Figure S1A** were normalized to the common peak at 1443 cm^{-1}. They combine the characteristic peaks of the starting materials, PBI and PEDOT:PSS with GOPS, confirming that the coating was successful for both spin and dip coated samples. The intensity of the PEDOT:PSS:GOPS peaks was higher for the spectrum of the PBI fibers modified by dip coating than for the ones modified by spin coating. Therefore, as the spectra **z**, **a** and **b** of **Figure S1A** were normalized to the intensity of the peak at 1443 cm^{-1} (ascribed to PBI), we may clearly conclude that the amount of PEDOT:PSS is larger in cross-linked PEDOT:PSS-dip coated PBI fibers. This result is consistent with the SEM results showing that the average diameter of dip coated fibers is larger than those modified by spin coating.

TGA studies. The TGA results revealed a significant reduction of the thermal stability of the PBI fibers upon coating with PEDOT:PSS:GOPS (**Figure S1B** and **S1C**, and **Table S1**). The mass loss of neat PBI fibers is negligible up to ca. 400 °C, where a significant degradation starts, stabilizing at ca. 500 °C with a residue of 12.3% of the initial mass. The first derivative indicates that the mass loss is maximum at 464 °C. The coating of the PBI fibers with PEDOT:PSS:GOPS reduces the thermal stability of the PBI/PEDOT composite. Mass loss starts at ca. 150 °C, with a more pronounced loss above ca. 400 °C (onset of the main peak in the first derivative, **Figure S1C**) as observed in the pristine PBI fibers. The increased in mass loss percentage at temperatures up to ca. 400 °C is attributed to the degradation of the PEDOT coating. A higher mass loss percentage, within this temperature range, was observed for the PEDOT:PSS-dip coated sample than for the PEDOT:PSS-spin coated sample; which is consistent with a relatively larger amount of PEDOT:PSS on the former samples.

The maximum mass loss rate (maximum of the first derivative) for pristine PBI and PEDOT:PSS dip coated PBI fibers occurs at similar temperatures (ca. 464 °C), though the mass loss occurs over a wider temperature range for the PEDOT:PSS-dip coated PBI fibers. The profile of mass loss in the PEDOT:PSS-spin coated samples, instead, shows a maximum mass loss rate at higher temperatures (ca. 500 °C). This results points to a slightly higher thermal stability of the PEDOT:PSS-spin coated samples, which we attribute to a higher efficiency of the cross-linking process. A possible cause is the difference in the GOPS content of the material adsorbed at the PBI fibers mat. In spin coating, we expect that the material making the PEDOT:PSS-coating has the same composition as the bulk composition of the dispersion, being the amount of material adsorbed controlled by the dispersion viscosity, the spinning speed and the surface adhesion of the dispersion materials. In the dip coating process, the adsorption depends on the interactions between the material in the suspension and the surface of the fibers. In view of the acidic nature of the PEDOT:PSS:GOPS dispersion (pH ≈ 1 - 2) in combination with the 24 h immersion time, we expect that protonation of PBI amine groups will occur at larger extent enhancing the adsorption of the negatively charged PSS via electrostatic interactions. In a simplistic picture, this would, in turn, promote the adsorption of the doped PEDOT chains, following a mechanism similar to that described by Decher et al. [5]. In our case though, we do not expect a sequential deposition because the positive and negatively charged polymers are entangled in the dispersion. It is the deposition of the material as "bundles" that ensures that the additives, namely the GOPS, are also adsorbed. However, there may be a lower GOPS content in the adsorbed

film when comparing to the content of the dispersion bulk, which would, in turn, lead to a loser network formation in the film. We believe this could explain the lower thermal stability of the dip coated samples when compared to the spin coated ones.

Sample	*PBI*	*PEDOT:PSS/PBI spin coating*	*PEDOT:PSS/PBI dip coating*	*PEDOT:PSS:GOPS pellet*
***Onset T1* (° C)**			124.8	152.8
***Peak T1* (° C)**			181.1	185.9
Weight Loss T1 (%)			8.5	12.2
***Onset T2* (° C)**		193.7	281.2	230.6
***Peak T2* (° C)**		275.7	333.2	301.9
Weight Loss T2 (%)		12.1	12.4	13
***Onset T3* (° C)**	373.6	353.4	376.3	337.1
***Peak T3* (° C)**	463.7	506.8	476.5	359.2
Weight Loss T3 (%)	81.1	76.6	71.6	4.7
***Onset T4* (° C)**				393
***Peak T4* (° C)**				416.8
Weight Loss T4 (%)				4.5
***Onset T5* (° C)**				440
Peak T5 (°C)				529.7
Weight Loss T5 (%)				47.5
Residue (%)	12.3	11.6	7.6	18

Table S1: TGA data compilation for the tested PEDOT:PSS/PBI fibers, and respective controls of PBI fibers and PEDOT:PSS pellet with GOPS, containing onset/peak temperature degradation (° C) and corresponding weight loss (w/w%).

2. Contact angle measurements

2.1. Materials and Methods

Contact angle measurements were performed through the sessile drop method using a Kruss DSA25B goniometer. PEDOT:PSS thin films cross-linked with GOPS were used as comparison. Those films were prepared by spin coating the aqueous PEDOT:PSS-coating dispersion (see Section 2.1) onto a clean glass coverslips as described in Section 2.3. The films obtained were annealed at 150 °C for 2 min. A drop of 2 μL of glycerol (Sigma-Aldrich) was deposited on the surface of the different fiber mats and films prepared (n = 7 independent samples per condition) and the Drop Shape Analysis 4 software was used to take measurements every 5 s for 2 min.

2.2. Brief Results and Dicussion

Contact angle analysis with glycerol was performed to assess the wettability of the nanofibrous scaffolds. The results of the analysis are shown in **Figure S2.**

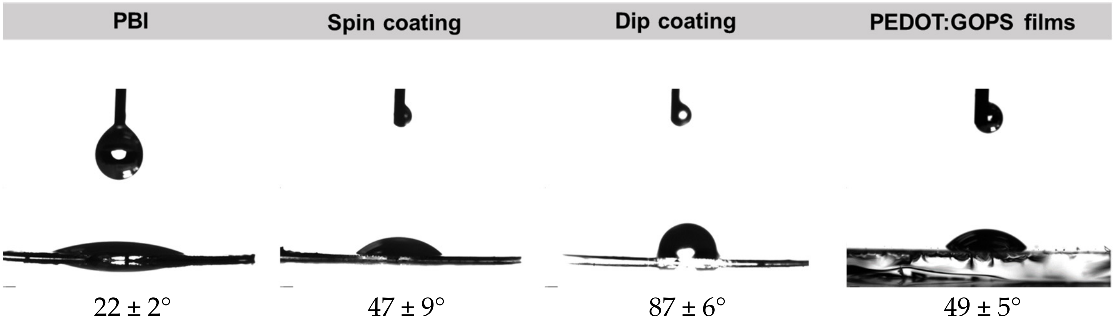

Figure S2. Contact angle analysis of pristine electrospun PBI fibers, cross-linked PEDOT:PSS-spin coated PBI electrospun fibers, cross-linked PEDOT:PSS-dip coated PBI electrospun fibers and cross-linked PEDOT:PSS films. The corresponding values of the contact angles (expressed as mean ± standard deviation (n=7 independent samples)) are shown below each photograph.

Pristine PBI nanofibers were shown to be highly hydrophilic, with a contact angle of 22 ± 2°. This value is lower than the value of 55 ± 5° measured in a flat PBI film, that is, the nanofibers mat is more hydrophilic than the flat PBI film despite its nanostructured surface. Flat films of cross-linked PEDOT:PSS show a contact angle of 49 ± 5°, approaching the value measured in PBI films. The coating of the PBI nanofibers with PEDOT:PSS, by either spin or dip coating, leads to surfaces that are more hydrophobic than the surface of pristine PBI fibers. More specifically, the samples modified by spin coating show a contact angle of 47 ± 9° while for those modified by dip coating the contact angle is 87 ± 6°. The contact angle of the PBI modified by spin coated PEDOT:PSS is similar to that of the flat cross-linked PEDOT:PSS films, while the high contact angle of the sample by dip coating is likely related to differences in the surface chemistry as a result of a more selective materials adsorption process, as discussed above. The details of the phase separation (between PEDOT and PSS-rich domains) will be different from those present in spin-coated films.

Surface wettability of the scaffold has a strong impact on stem cell behavior [6]. Kim et al. [7] have studied the response of hBMSCs on polymer surfaces with different wettability and they found that the cell adhesion was significantly higher on highly hydrophilic and rough surfaces than in hydrophobic and smooth surfaces. In particular, Hao et al. [8] reported that the adhesion and spreading of hMSCs is enhanced by highly hydrophilic substrates, while moderate hydrophilic

surfaces promoted osteogenic differentiation. The surface properties of both our PEDOT:PSS-coated PBI scaffolds can potentially benefit MSC proliferation and still be suitable for other biomedical applications.

3. References

1. Lee, S.; Park, H.; Son, W.; Choi, H.H.; Kim, J.H. Novel solution-processable, dedoped semiconductors for application in thermoelectric devices. *J. Mater. Chem. A*., **2014**, *2*, 13380–13387. doi: 10.1039/C4TA01839G
2. Zhao, Q.; Jamal, R.; Zhang, L.; Wang, M.; Abdiryim, T. The structure and properties of PEDOT synthesized by template-free solution method. *Nanoscale Res. Lett.*, **2014**, *9*, 557. doi:10.1186/1556-276X-9-557
3. Šapić, I.M.; Bistričić, L.; Volovšek, V.; Dananić, V. Vibrational Analysis of 3-Glycidoxypropyltrimethoxysilane Polymer. *Macromol. Symp.*, **2014**, *339*, 122-129. doi: 10.1002/masy.201300145.
4. Vale, M.; Loureiro, M.V.; Ferreira, M.J.; Marques, A.C. Silica-based microspheres with interconnected macroporosity by phase separation. *J. Sol-Gel Sci. Technol.*, **2020**, *95*, 746–759. doi: 10.1007/s10971-020-05257-4
5. Decher, G.; Hong, J. D.; Schmitt, J. Buildup of ultrathin multilayer films by a self-assembly process: III. Consecutively alternating adsorption of anionic and cationic polyelectrolytes on charged surfaces. *Thin Solid films*, **1992**, *210-211*, 831-835. doi: 10.1016/0040-6090(92)90417-A
6. Li, Y.; Wang, S.; Jiang, L. Chapter 16 - Wettability Effect on Stem Cell Behavior. In Biology and Engineering of Stem Cell Niches, Vishwakarma, A., Karp, J.M. (Eds), Elsevier, 2017, 16, pp. 245-255, doi 10.1016/B978-0-12-802734-9.00016-0. ISNB: 978-0-12-802734-9.
7. Kim, M.S.; Shin, Y.N.; Cho, M.H.; Kim, S.H.; Kim, S.K.; Cho, Y.H.; Khang, G.; Lee, I.W.; Lee, H.B. Adhesion Behavior of Human Bone Marrow Stromal Cells on Differentially Wettable Polymer Surfaces. *Tissue Eng.*, **2007**, *13*, 2095-2103. doi: 10.1089/ten.2006.0062
8. Hao, L.; Yang, H.; Du, C.; Fu, X.; Zhao, N.; Xu, S.; Cui, F.; Mao, C.; Wang, Y. Directing the fate of human and mouse mesenchymal stem cells by hydroxyl–methyl mixed self-assembled monolayers with varying wettability. *J. Mater. Chem. B*, **2014**, *2*, 4794-4801. doi: 10.1039/C4TB00597J
